# Supplementary material for: Description of a Cohort with a New Truncating MYBPC3 Variant for Hypertrophic Cardiomyopathy in Northern Spain
Source: Genes (Basel). 2023 Mar 30;14(4):840. doi: 10.3390/genes14040840 (PMC10137663; doi:10.3390/genes14040840)
Supplement: Supplementary file 1 [file genes-14-00840-s001.zip › genes-2249714-supplementary.pdf]

Supplementary Figure S1. Sanger sequencing analysis of III-1 member from family 8, according p.Val931Glyfs*120 MYBPC3 variant.

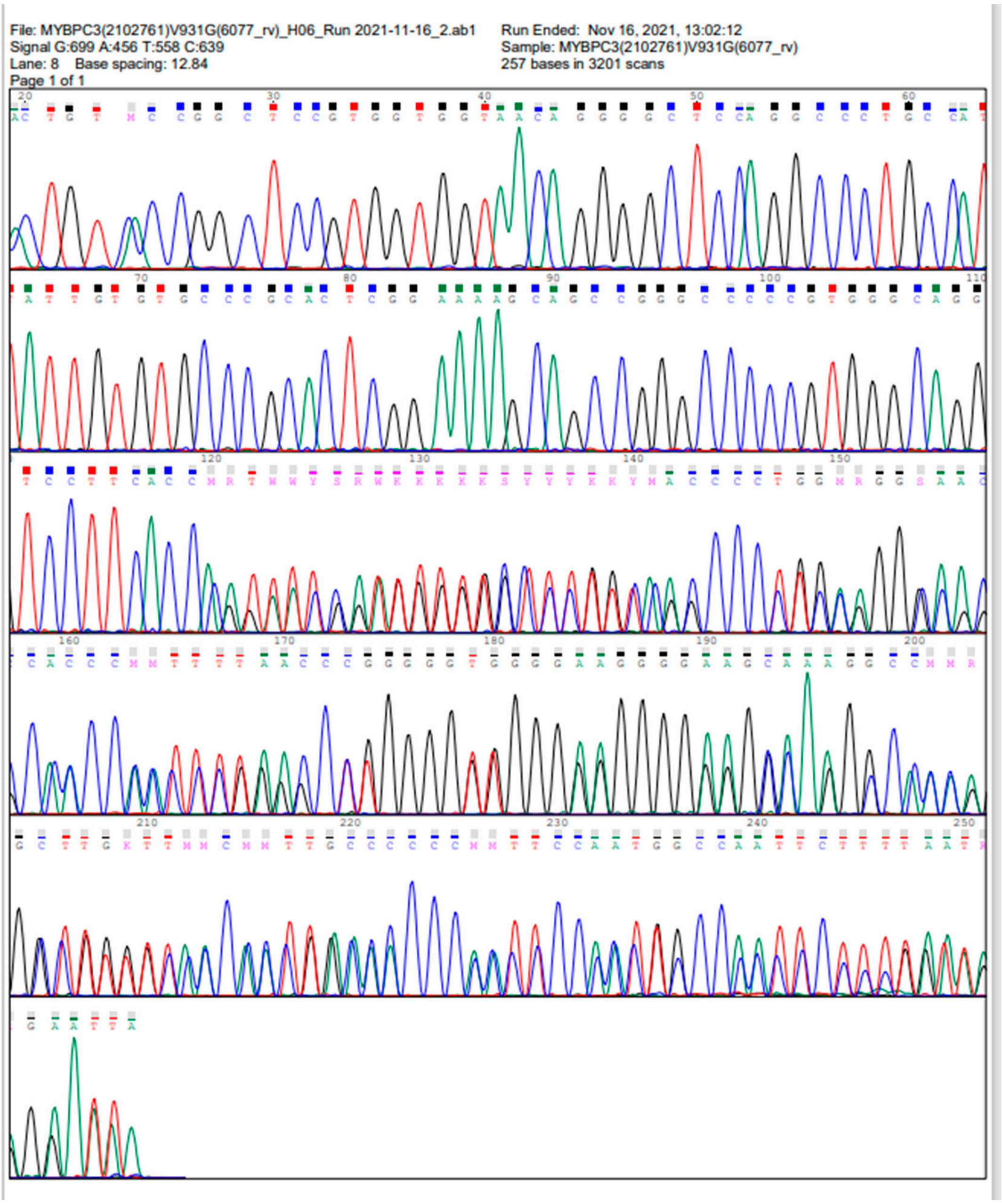

Sanger sequencing was performed with a custom primer design that covers the specific region of the genome where the variant is located, both forward and reverse. Sequencing was performed in both forward and reverse (data shown) using the SeqStudio Genetic Analyzer System.

Insertion of a G base, approximately at position 120, of the amplified sequence (red arrow) generates a downstream frameshift alteration, which generate the formation of a STOP codon 120 amino acids downstream from the insertion.
